# Practical Observations on the Causes and Treatment of Curvatures of the Spine

**Published:** 1840-01

**Authors:** 


ART. VI.—*Practical Observations on the Causes and Treatment of Curvatures of the Spine.* By SAMUEL HARE, Surgeon.—*London and Leeds,* 1838. 8vo, pp. 151.

THIS is a handsome volume; and the plates, exhibiting, it is said, exact portraits of various incurvations and the ameliorations produced by treatment, are really beautiful. Mr. Hare's book is, however, by no means a scientific one, and claims little notice at our hands. It contains a description of certain machinery for the remedying of spinal curvatures, which is said to prove most serviceable in the author's hands, and narrates a few cases which attest the success of Mr. Hare's particular management. We cannot, however, say that the work before us has added anything of the least value to our previous knowledge.

